# Fever Nursing

**Published:** 1908-03-07

**Authors:** 


### Nursing Administration.

### FEVER NURSING.

### II.—THE NEED FOR ORGANISATION.

The recent advances in scientific fever nursing have placed the fever nurse in a distinctly ambiguous position. It is not merely, as with mental nursing, that the value of her training depends altogether on the character of the institution in which it has been received. It is that the circumstances under which knowledge of fever nursing can be picked up are curiously irregular. We say "picked up" because some of the largest employers of fever nurses do not profess to give any regular training at all to their employees. There are three methods open to the woman who desires to enter for this branch of work. It is possible for her to enter upon an engagement of a few months in a fever hospital, use her wits, and depart at the end of the shortest period which entitles her to a testimonial, and this period is, we believe, three months. During this time she has grown accustomed to wearing a uniform, has, perhaps, learned how to take a temperature, how to wash a patient, how to administer medicines and sponge the eyes of a child ill with measles. She has also learned the use of disinfectants, and some of the ordinary precautions to be observed in nursing infectious diseases. She will, therefore, feel justified in posing as a fever nurse, and such is the congested state of the calling that in times of epidemic she may be pretty sure of securing an engagement at will. The books of the smaller registry-offices are open to these "nurses" who have had what by courtesy they describe as a little fever training, and there is no doubt that after taking a few cases at very low fees, they often obtain admission to nursing homes, and so blossom out into full-blown "pseudo-nurses."

The more regular method of obtaining instruction in fever nursing is to enter an institution for a two years' course. It may or may not be training in the technical sense of the word, implying attendance at lectures and the passing of an examination. In by far the greater number of fever hospitals, including many of those under the Metropolitan Asylums Board, little attempt is made to encourage serious study among the assistant nurses, and the mere fact that they are not accorded the name of probationers is in itself significant. Moreover, the system under which assistant nurses are changed from one hospital to another during temporary lulls in the work tends to neutralise the matron's influence and make it difficult for a high tone to be maintained. In the hospitals of the Metropolitan Asylums Board the nurses do not receive a certificate, even after their two years' course, although they are entitled to a testimonial should their conduct have proved satisfactory. It is evident, therefore, that in many institutions it depends almost entirely on the nurse herself whether at the end of her two years' course she has become efficient. In no branch of nursing is there a wider difference in the quality of the training afforded as between one institution and another.

Should she desire to obtain general training a fever nurse needs to have a particularly good record. The matron of a Poor-law Infirmary or of a small general hospital requires evidence that the nurse she is to receive for one year is distinctly promising in ability and disposition. An average nurse has little chance of getting this coveted year's training, for the applications for it are numerous, and the best will be picked. Here again, then, is a possible source of danger to the nursing profession. A certain number of regularly trained fever nurses, and these the least efficient of their class, are free at the end of two years to undertake nursing on their own account without a vestige of general training. It might be deemed reasonable that these women should form an efficient reserve in times of epidemics and prove a barrier against the admission to the fever hospitals of the class of persons we have described above, whose fever training is merely nominal. It does not appear, however, that any considerable number of two-year fever trained nurses is ever available for emergencies. Probably the fees offered are insufficient and the work too precarious. Whatever the reason, it is generally admitted that to advertise for fever nurses in an epidemic brings together a somewhat motley crew. The two-year trained fever nurse is barred, quite rightly, from promotion in her own hospital unless she can obtain general training, and the outlets she obtains for her energies can only be imagined.

The third, and for the nurse herself the best, manner of receiving fever training is to go on to a fever hospital at the conclusion of her three years' general training or during her third year. Indeed, some medical superintendents of these institutions where experimental work in the direction of checking infection is incessantly going on, have testified to the great debt which they owe in their researches to the enthusiasm of the hospital nurses who have become attached to this branch of fever nursing. Unhappily, it is impossible to conduct all fever training on these principles. However excellent the organisation, it has been proved that no hospital can allow the number of nurses under special instruction to rise above 10 per cent. And it requires a clever matron to arrange the ward routine to good advantage when one nurse out of every ten is a special pupil, received for a few months only and unattached to the regular staff. There is also a danger, where many such pupils are received, that the regular probationers may be deprived of the best work, in favour of new-comers.

It is fair to state that those who are engaged in training fever nurses concur in preferring that the fever training should be taken first.

Thus, the making of fever nurses is exposed to many disadvantages, and the need for better organisation, for some authoritative recognition of the good training, and some definite steps with regard to the bad, is growing imperative.

